# Comparative analysis of neutrophil to lymphocyte ratio and derived neutrophil to lymphocyte ratio with respect to outcomes of in-hospital coronavirus disease 2019 patients: A retrospective study

**DOI:** 10.3389/fmed.2022.951556

**Published:** 2022-07-22

**Authors:** Muhammad Sohaib Asghar, Mohammed Akram, Farah Yasmin, Hala Najeeb, Unaiza Naeem, Mrunanjali Gaddam, Muhammad Saad Jafri, Muhammad Junaid Tahir, Iqra Yasin, Hamid Mahmood, Qasim Mehmood, Roy Rillera Marzo

**Affiliations:** ^1^Department of Internal Medicine, Dow University Hospital, Karachi, Pakistan; ^2^Department of Internal Medicine, Liaquat National Hospital and Medical College, Karachi, Pakistan; ^3^Department of Internal Medicine, Dow University of Health Sciences, Karachi, Pakistan; ^4^Department of Internal Medicine, The Brooklyn Hospital Center, Brooklyn, NY, United States; ^5^Department of Internal Medicine, Ziauddin University Hospital, Karachi, Pakistan; ^6^Department of Internal Medicine, Lahore General Hospital, Lahore, Pakistan; ^7^Department of Internal Medicine, King Edward Medical University, Lahore, Pakistan; ^8^Department of Community Medicine, International Medical School, Management and Science University, Shah Alam, Malaysia; ^9^Global Public Health, Jeffrey Cheah School of Medicine and Health Sciences, Monash University, Subang Jaya, Malaysia

**Keywords:** NLR, COVID-19, management, severity, marker

## Abstract

**Introduction and objectives:**

In patients with coronavirus disease 2019 (COVID-19), several abnormal hematological biomarkers have been reported. The current study aimed to find out the association of neutrophil to lymphocyte ratio (NLR) and derived NLR (dNLR) with COVID-19. The objective was to compare the accuracy of both of these markers in predicting the severity of the disease.

**Materials and methods:**

The study was conducted in a single-center having patients with COVID-19 with a considerable hospital stay. NLR is easily calculated by dividing the absolute neutrophil count (ANC) with the absolute lymphocyte count (ALC) {ANC/ALC}, while dNLR is calculated by ANC divided by total leukocyte count minus ANC {ANC/(WBC-ANC)}. Medians and interquartile ranges (IQR) were represented by box plots. Multivariable logistic regression was performed obtaining an odds ratio (OR), 95% CI, and further adjusted to discover the independent predictors and risk factors associated with elevated NLR and dNLR.

**Results:**

A total of 1,000 patients with COVID-19 were included. The baseline NLR and dNLR were 5.00 (2.91–10.46) and 4.00 (2.33–6.14), respectively. A cut-off value of 4.23 for NLR and 2.63 for dNLR were set by receiver operating characteristic (ROC) analysis. Significant associations of NLR were obtained by binary logistic regression for dependent outcome variables as ICU stay (*p* < 0.001), death (*p* < 0.001), and invasive ventilation (*p* < 0.001) while that of dNLR with ICU stay (*p* = 0.002), death (*p* < 0.001), and invasive ventilation (*p* = 0.002) on multivariate analysis when adjusted for age, gender, and a wave of pandemics. Moreover, the indices were found correlating with other inflammatory markers such as C-reactive protein (CRP), D-dimer, and procalcitonin (PCT).

**Conclusion:**

Both markers are equally reliable and sensitive for predicting in-hospital outcomes of patients with COVID-19. Early detection and predictive analysis of these markers can allow physicians to risk assessment and prompt management of these patients.

## Introduction

The coronavirus disease 2019 (COVID-19) continues to jeopardize humanity and challenge modern healthcare (1). As of January 2022, there are more than 300 million reported cases, while the death toll has surpassed 5 million globally (2). The severe acute respiratory syndrome coronavirus 2 (SARS-CoV-2) is the pathogen of this atypical pneumonia outbreak which targets the lower respiratory tract, predisposing to multiple organ involvement through the distribution of angiotensin-converting enzyme 2 (ACE-2) (1). The main mode of transmission is person to person by the respiratory droplets coughed or sneezed by the infected person being inhaled by other people in close vicinity. SARS-CoV-2 can remain stable and infectious in aerosols for hours, and if present on surfaces and objects touched by a non-infected person who later touches his eyes, nose, or mouth can also be a possible route of spread (3). Declared by WHO as a public health emergency (4), the disease progression has been marked by the appearance of different variants. These display increased transmissibility, severe disease course, reduced effectiveness of treatments, and each wave is signified by a new “Variant of Concern (VOC).” After the Delta variant, the Omicron variant is now the VOC and is a heavily mutated form of SARS-CoV-2 (5).

The symptoms of COVID-19 can be classified into ordinary, mild, severe, and critical (4), and the disease presents with a wide range of clinical manifestations from asymptomatic to symptomatic, namely, respiratory symptoms, fever, shortness of breath, cough, dyspnea, and viral pneumonia and in severe cases, pneumonia, severe acute respiratory syndrome, heart failure, renal failure, and even death (6). With the number of instances climbing daily, hospitals worldwide are faced with an influx of patients with COVID-19 (7). In these pressing circumstances, identifying patients early and ascertaining whoever is at a higher risk of death to better manage and allocate resources (7) is imperative. A pragmatic risk stratification tool can be utilized in these situations, facilitating appropriate and timely intervention (7). Previously, markers of inflammation have been found to prognosticate patients successfully (1). In SARS-CoV-2, several abnormal hematological biomarkers have been reported (8). The parameters used to assess and stratify the risk category of patients with COVID-19 include white blood cell (WBC) count, lymphocyte count, neutrophil count, neutrophil-lymphocyte ratio (NLR), derived NLR ratio (d-NLR), platelet count, eosinophil count, hemoglobin, D-dimer, and fibrinogen levels (8–10).

Among the markers enlisted, NLR is found to have been previously useful for prognosticating in conditions such as sepsis, cardiovascular diseases, and malignant tumors (8). It is a relatively new biomarker for determining systemic inflammation (11) and is the ratio of absolute neutrophil count (ANC) to absolute lymphocyte count (ALC) (12), where a high NLR value is indicative of a high neutrophil count but a decreased lymphocyte count (11). In addition, d-NLR is the ratio of ANC to the difference between WBC count and ANC (10). Both, NLR and d-NLR are raised in chronic conditions with a low-grade inflammatory nature, such as obesity, hypertension, diabetes mellitus, metabolic syndrome, atherosclerotic events of the heart and brain, and several cancers. These illnesses are deemed as risk factors for COVID-19 (1). Various studies reporting COVID-19 have found NLR to discern between mild/moderate and severe/critical groups and the probability of death in patients with COVID-19 infection (13–21). Investigations have evaluated the significance of NLR in predicting progression to severe disease, risk of intubation, risk of severe disease in intubated patients, days intubated, intensive care unit (ICU) admission, and longer ICU stay (1), and an elevated NLR is also correlated with mortality (13).

In the current scenario of the pandemic where concurrent ailments are presented in patients, the risk of death changes over time, and the pressure on the healthcare system mounts (7). In our study, we evaluated NLR and d-NLR in patients with COVID-19 and assessed their effectiveness as biomarkers for screening and clinical management of COVID-19 (9). We aim to study the association of these markers with the identification of crucial and high-risk patients, to prevent significant progression of COVID-19 and predict outcomes coupled with the infection.

## Materials and methods

**FIGURE 1:**
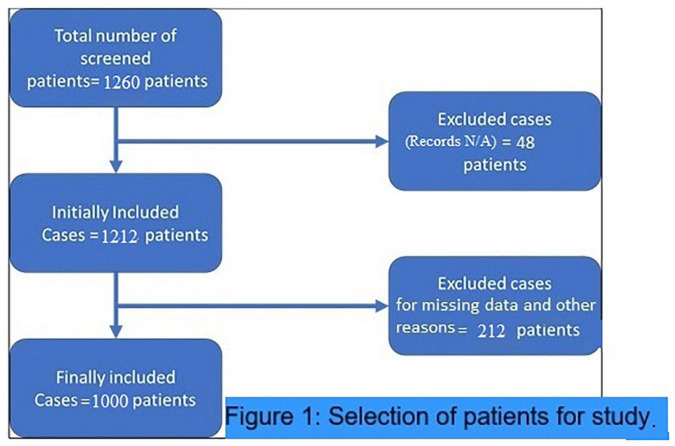
Patient recruitment flow chart for the study.

This retrospective study was conducted in a single center having patients with COVID-19 to find out the association of comparatively simple, inexpensive, and practical severity markers of COVID-19. These hematological indices are NLR and dNLR. NLR is easily calculated by dividing ANC with (ALC) {ANC/ALC}, while dNLR is calculated by dividing ANC with WBC count minus ANC {ANC/(WBC–ANC)}. The baseline laboratory values on the admission of these markers were considered for the analysis to correlate with the probability of worse outcomes. There were no specific criteria for inclusion of the patients except for those who were discharged for home quarantine before complete recovery, or either had missing data and unavailable medical records were excluded. Since 59% of the patients stayed in intensive care during their hospital stay and approximately 30% of them died during their disease course. The ICU admission and mortality rates were obviously higher than that of common patients with COVID-19. Thus, the population investigated in this study had a definite higher hospital stay considerable for inclusion in the study to have complete data available as shown in patient selection criteria (Figure 1). All samples were analyzed for complete blood picture using the CELL-DYN Ruby Hematology Analyzer (Abbott Laboratory, IL, United States) which is an automated multiparameter design utilizing multiangle polarized scatter separation (MAPSS) technology to determine the cell count analysis.

The descriptive data of the study participants included age; gender; pandemic wave-first, second, or third; ICU stay; the outcome as recovery, home quarantined or death; mode of respiration during the hospital stay; duration of diagnosis of COVID-19 infection; and duration of hospital stay. Medians and interquartile ranges (IQRs) were represented by box plots. Multivariable logistic regression was performed obtaining odds ratio (OR), 95% CI, and further adjusted to discover the independent predictors and risk factors associated with elevated NLR and dNLR. A *p*-value was considered significant if less than 0.05 (two-tailed). Linear correlation of studied markers was performed with other markers of inflammatory response like C-reactive protein (CRP), procalcitonin (PCT), and D-dimer levels to report Spearman’s rho (*r*). Furthermore, receiver operating characteristic (ROC) analysis was conducted to determine the predictability of these markers for different outcomes of the disease. An optimum cut-off was obtained by using an appropriate Youden index as a summary measure for the area under the curve (AUC). Subsequently, a 2 × 2 contingency table was plotted to find out appropriate sensitivity, specificity, positive predictive value (PPV), negative predictive value (NPV), positive likelihood ratio (+) LR, and negative likelihood ratio (–) LR, and accuracy along with their standard error. Since there was a linear relationship between both the parameters, a parallel comparison of ROC curves was performed *via* Delong’s test.

## Results

### Baseline statistics

A total of 1,000 patients was included in the final analysis, the majority of whom were recruited from the first (36.8%) and the third waves of the pandemic (37.9%). Of the total patients enrolled, 68% were men, 59% were 51–75 years old, 59% stayed in intensive care during their hospital stay, and approximately 30% died during the course of the disease. The frequent modes of ventilation were BiPAP (21%), oxygen by face mask (20%), and invasive ventilators (19%). Another 21% did not require any form of supplemental oxygen during their hospital stay. As shown in Table 1, the median days of stay were 6.00 (3.00–10.00), the neutrophil count was 80.00% (70.00–86.00%), the ANC was 7.82 (4.82–11.91), the lymphocytes were 16.00% (8.00–24.00%), the ALC was 4.00 (3.00–6.00), the absolute monocyte count was 1.42 (0.90–2.01), the NLR was 5.00 (2.91–10.46), and the dNLR was 4.00 (2.33–6.14).

**TABLE 1:** Baseline data of the study subjects (*n* = 1000).

Variables	Characteristics	Median/Frequency (%)	IQR/95% confidence interval of percentages
Age groups	<25 years	27 (2.7)	1.8–4.0%
	26–50 years	305 (30.5)	27.6–33.5%
	51–75 years	596 (59.6)	56.4–62.7%
	>75 years	72 (7.2)	5.7–9.0%
Gender	Male	681 (68.1)	65.1–71.0%
	Female	319 (31.9)	29.0–34.9%
Patient selection	First wave	368 (36.8)	33.8–39.9%
	Second wave	253 (25.3)	22.6-28.1%
	Third wave	379 (37.9)	34.9–41.0%
Hospital stay	Non-ICU	408 (40.8)	37.7–43.9%
	ICU	592 (59.2)	56.1–62.3%
Comorbidities	DM	422 (42.2)	35.0–49.6%
	HTN	546 (54.6)	47.1–61.9%
	COPD	16 (1.6)	0.3–4.7%
	CKD	103 (10.3)	6.3–15.7%
	CAD	104 (10.4)	6.4–15.7%
	CLD	11 (1.1)	0.1–3.9%
	Chronic viral hepatitis	16 (1.6)	0.3–4.7%
	Asthma	43 (4.3)	1.9–8.4%
	Tuberculosis	5 (0.5)	0.0–3.0%
Patient outcome	Recovered	522 (52.2)	49.1–55.3%
	Self-quarantined	179 (17.9)	15.6–20.4%
	Death	299 (29.9)	27.1–32.8%
Mode of respiration	None	213 (21.3)	18.8–24.0%
	Invasive (ventilator)	190 (19.0)	16.6–21.6%
	BiPAP	210 (21.0)	18.5–23.7%
	CPAP	41 (4.1)	3.0–5.5%
	Oxygen mask	202 (20.2)	17.8–22.8%
	Nasal cannula	144 (14.4)	12.3–16.7%
Duration of diagnosis of COVID-19 infection	(in days)	14.00	10.00–17.00
Duration of hospital stay	(in days)	6.00	3.00–10.00
CRP	(mg/L)	17.98	5.87–25.24
D-dimer	(mcg/mL)	1.64	0.73–6.00
PCT	(ng/dL)	0.51	0.15–1.94
Total leukocyte count	(× 10^9^/liter)	9.90	6.90–14.20
Differential leukocyte count	Neutrophils (%)	80.00	70.00–86.00
	Lymphocytes (%)	16.00	8.00–24.00
	Monocytes (%)	4.00	3.00–6.00
Absolute neutrophil count	(× 10^9^/liter)	7.82	4.82–11.91
Absolute lymphocyte count	(× 10^9^/liter)	1.42	0.90–2.01
Neutrophil to lymphocyte ratio (NLR)	Absolute neutrophil count/Absolute lymphocyte count	5.00	2.91–10.46
Derived neutrophil to lymphocyte ratio (dNLR)	Absolute neutrophil count/(Total leukocyte count – Absolute neutrophil count)	4.00	2.33–6.14

Data presented as either median (IQR) or frequency (%).

IQR, interquartile range; ICU, intensive care unit; BiPAP, bilevel positive airway pressure; CPAP, continuous positive airway pressure; COVID-19, coronavirus disease 2019; CRP, C-reactive protein; PCT, Procalcitonin; DM, diabetes mellitus; HTN, hypertension; COPD, chronic obstructive pulmonary disease; CKD, chronic kidney disease; CAD, coronary artery disease; CLD, chronic liver disease.

### Association of neutrophil to lymphocyte ratio and derived neutrophil to lymphocyte ratio with disease outcomes

**FIGURE 2:**
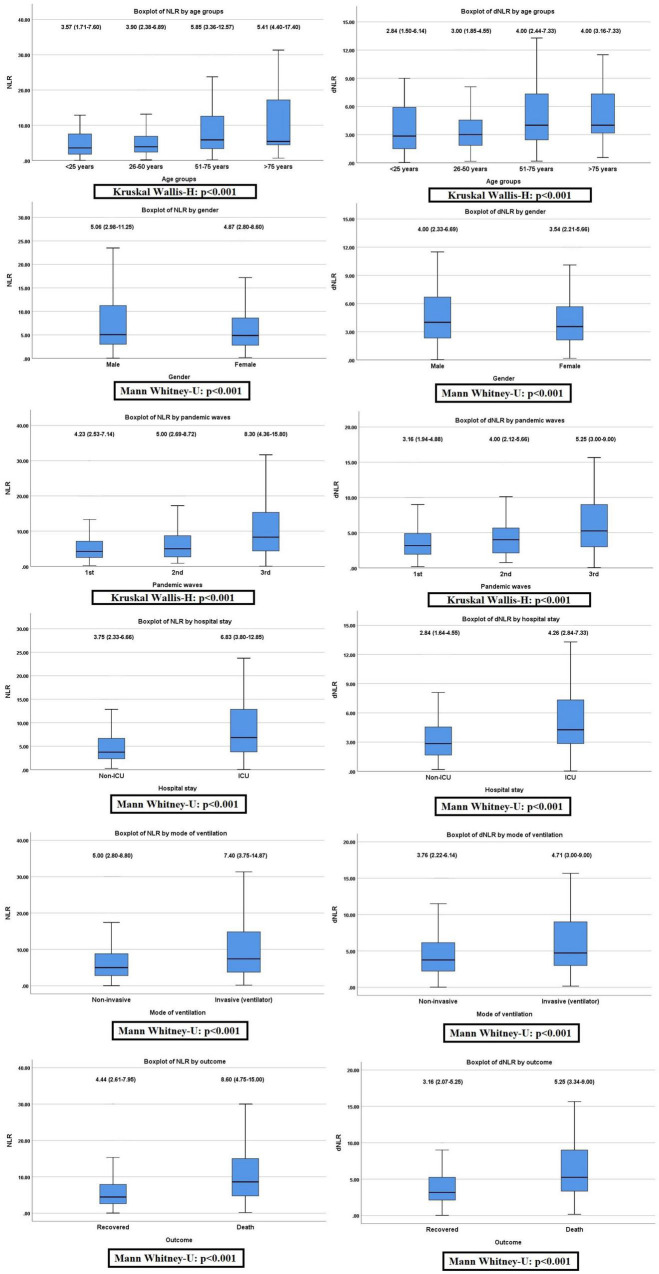
Non-parametric distribution of neutrophil to lymphocyte ratio (NLR) and derived NLR (dNLR) among the study variables (Mann–Whitney *U*-test or Kruskal–Wallis *H*-test applied as indicated).

**FIGURE 3:**
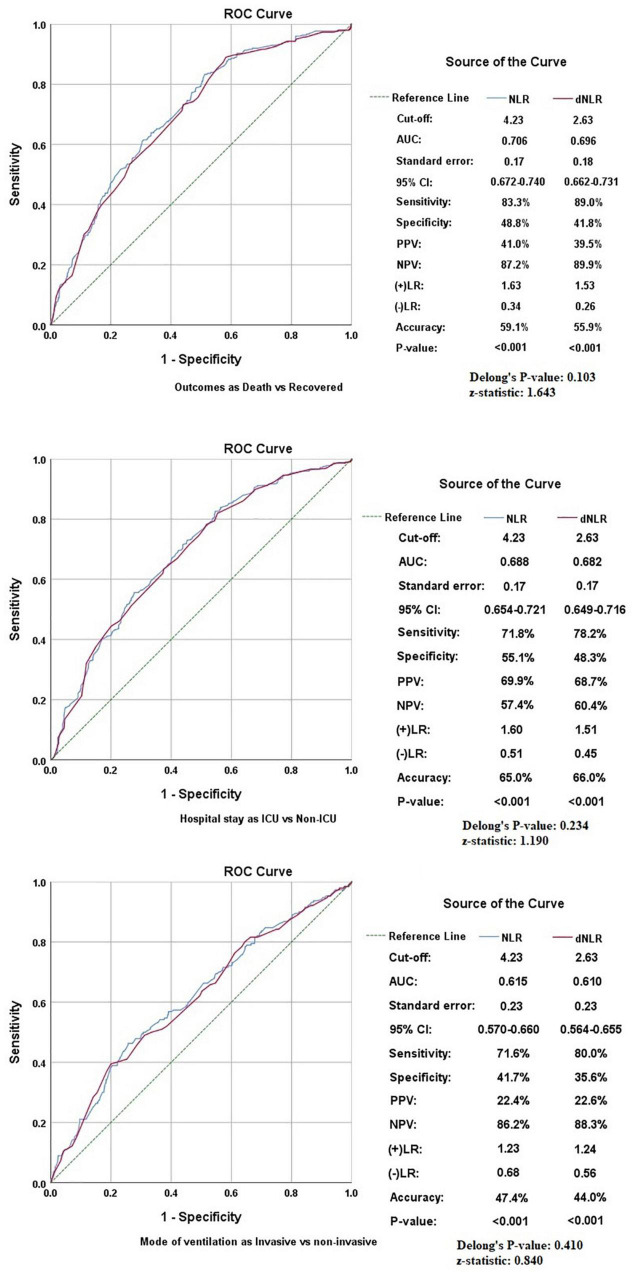
Receiver operating characteristics (ROCs) showing the association of neutrophil to lymphocyte ratio (NLR) and derived NLR (dNLR) with study variables.

The pattern of distribution of both these biomarkers among age, gender, pandemic waves, ICU stay, invasive ventilation, and survival outcome is shown in Figure 2. Receiver operating analysis obtained a cut-off of 4.23 for NLR and 2.63 for dNLR as shown in Figure 3. Univariate analysis demonstrated that the probability of higher NLR was among the 51–75 years of age group (*p* < 0.001), >75 years (*p* < 0.001), ICU stay (*p* < 0.001), invasive ventilation (*p* = 0.001), death (*p* < 0.001), and the second wave (*p* = 0.026) and the third wave of pandemic (*p* < 0.001), as shown in Supplementary Figure 1. Similarly, univariate analysis revealed that patients in the 51–75 year of age group (*p* = 0.001), >75 years (*p* = 0.001), males (*p* = 0.043), ICU stay (*p* = 0.001), invasive ventilation (*p* = 0.001), death (*p* = 0.001), and the third wave of pandemic (*p* = 0.001) had a higher dNLR. On multivariable and survival analysis, age, ICU stay, invasive ventilation remain independent predictors as shown in Table 2 and Supplementary Figure 2.

**TABLE 2:** Binary logistic regression of neutrophil to lymphocyte ratio (NLR) and derived NLR (dNLR) as continuous variables with the study outcomes.

Variables		ICU stay vs. ward	Death vs. recovery	Invasive vs. non-invasive ventilation
NLR	Univariate	1.060 [1.039–1.081]*	1.064 [1.046–1.082]*	1.034 [1.019–1.049]*
	Adjusted for age and gender	1.033 [1.013–1.052]*	1.041 [1.024–1.058]*	1.035 [1.019–1.052]*
	Further adjusted for waves of pandemic	0.991 [0.972–1.010]	1.040 [1.022–1.057]*	1.029 [1.012–1.046]*
dNLR	Univariate	1.168 [1.119–1.220]*	1.166 [1.124–1.210]*	1.091 [1.053–1.130]*
	Adjusted for age and gender	1.120 [1.067–1.174]*	1.111 [1.067–1.156]*	1.101 [1.056–1.148]*
	Further adjusted for waves of pandemic	1.011 [0.957–1.068]	1.106 [1.059–1.155]*	1.075 [1.028–1.125]*

Data presented as odds ratios (OR) for univariate analysis and adjusted odds ratio (aOR) for multivariate analysis {along with their 95% CI}.

*Denotes significance (*p* < 0.05) through Wald’s method.

NLR, neutrophil to lymphocyte ratio; dNLR, derived neutrophil to lymphocyte ratio; ICU, intensive care unit.

### Correlation statistics

**FIGURE 4:**
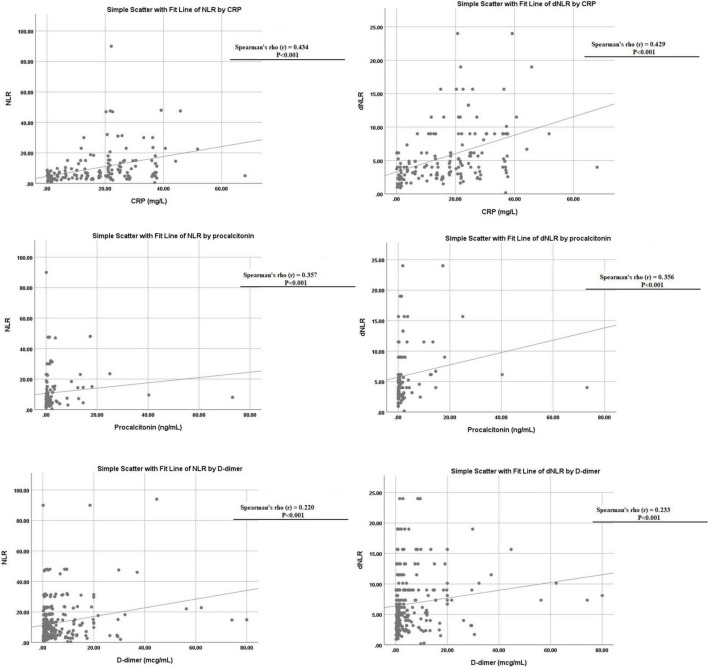
Linear correlation of neutrophil to lymphocyte ratio (NLR) and derived NLR (dNLR) with C-reactive protein (CRP), D-dimer, and procalcitonin levels.

Both NLR and dNLR were also found to correlate with other markers of inflammatory response like CRP (*p* < 0.001), PCT (*p* < 0.001), and D-dimer (*p* < 0.001), respectively, as shown in Figure 4.

### Comparative analysis among neutrophil to lymphocyte ratio and derived neutrophil to lymphocyte ratio, which is the better marker?

Although both markers had similar accuracy in predicting various outcomes in patients with COVID-19, some minor differences were found in our comparative analysis. First of all, dNLR was found to be higher in males while no significant difference was noted in NLR in patients of different genders. The AUC was slightly higher for NLR in all outcomes, but dNLR has shown slightly higher sensitivities at optimum cut-off corresponding with the higher negative likelihood ratios shown by NLR. Delong’s test applied to all the study variables showed indiscriminate significance among NLR and dNLR as shown in Figure 3. Furthermore, the linear relationship between NLR and dNLR was highly significant {Spearman’s correlation coefficient (rho): 0.964 (*p* < 0.001)}. Lastly, the odds-on multivariate analysis for dNLR was slightly higher in the >75 years of age group [aOR: 3.711 (1.715–8.029)] as compared to NLR [aOR: 2.499 (1.314–4.751)], and also in mortality [aOR: 4.418 (2.818–6.928)] vs. [aOR: 3.777 (2.545–5.604)] both being statistically significant. The odds for NLR were slightly higher in the second wave [aOR: 1.319 (0.936–1.858)] as compared to dNLR [aOR: 1.096 (0.771–1.559)], and also in the third wave [aOR: 2.148 (1.467–3.145) vs. aOR: 1.921 (1.279–2.884)] with only the latter being statistically significant.

## Discussion

The current study evaluated the evidence comprising a large cohort of individuals on the effectiveness of hematological markers NLR and dNLR in prognosticating patients and predicting outcomes in COVID-19 infection. Comprising patients over the first three waves of the pandemic, the highest number of included patients was from the first and third waves. Demographical data in our analysis was comparable to other large patient population studies, with 68% of the population in our analysis being male. The age group of our population was comprised mostly of people aged 51–75 years (59%). The death rate was unexceptional at 30% and was the lowest recorded patient outcome since most patients recovered (52%). The frequent mode of ventilation was BiPAP (21%), which is one of the two primary traditional non-invasive ventilation (NIV) (12), the other being continuous positive airway pressure (CPAP). These NIV methods have been used for up to 70% of patients with COVID-19 before tracheal intubation (12). A total of 20% of individuals in our study used oxygen by face masks, which can provide high FiO_2_ oxygen therapy (12). About 19% required an invasive ventilator, while another 21% did not require any form of supplemental oxygen during their hospital stay.

Coronavirus disease 2019 pathogenesis displays an immune imbalance, and a 16-day longitudinal analysis revealed that those undergoing a severe disease course showed a sustained increase in neutrophil count while the lymphocyte count displayed a unique dynamic that entailed a decrease in the first week but a progressive increase in the second week, a level same as that of mild patients (13) and no longer statistically significant (22). Acute respiratory distress syndrome (ARDS) and organ injury during COVID-19 infection correlate with the extensive lung infiltration of neutrophils and increased neutrophil counts in the peripheral blood of refractory patients, and an increase in neutrophil counts during the immunopathological phase of the infection is related to the inflammatory response’s intensity (13). The NLR depicts the inflammatory status of patients and contributes to better risk stratification in inflammatory and infectious diseases. The latest literature has established an NLR cut-off for the population in good health as being 0.78–3.53 (22).

A few studies evaluating outcomes predictions in COVID-19 investigated markers, namely, NLR, lymphocyte-to-CRP ratio, platelet-to-lymphocyte (PLR) ratio, and dNLR and revealed that NLR exhibited the highest specificity and severity for illness and was established as an independent predictor for mortality (13). The median NLR and dNLR were 5.00 (2.91–10.46) and 4.00 (2.33–6.14), respectively, in our analysis with a cut-off of 4.23 for NLR and 2.63 for dNLR. These findings are comparable to recent studies. A retrospective analysis performed during the months coinciding with the first wave of the pandemic evaluated the association of NLR role with the prediction of severe COVID-19 with an optimal cutoff of 4.795 (23). Sayed et al.’s overall analysis in their study deduced an NLR value of 5.5 which has a high prognostic and high specificity of 91.43 and 96.4%, respectively, indicating that a person with an NLR of 5.5 or more was likely to have a COVID-19 infection (24). Nasir et al. in an observational study at a tertiary care center showed that NLR ≥5 was independently associated with mortality (25). Furthermore, Chen et al. identified through the ROC analysis an NLR of 6.66 as the optimal cut-off to discriminate between discharge and death outcome (26). A single-centered study in Turkey determined an optimal cut-off value as NLR >3.69, when ROC analysis for the diagnosis power of age and laboratory values in the prognosis of the disease severity among all patients were examined (27). Tatum et al. in the analysis of the points of each ROC curve by maximizing Youden’s index revealed optimal NLR cut-off values of 9.96 for hospital day 2 and 11.40 for hospital day 5 (28). Fesih et al. determined the optimal cut-off value for NLR to be above 3.27, which estimated disease severity (29). In a retrospective study in Turkey, the ROC curve analysis was performed to distinguish the patients with COVID-19 from healthy controls. The optimal cut-off value for NLR was 3.58 (30). Güneysu et al. indicated that ROC analysis of the inflammatory markers was statistically significant in their retrospective study with a cut-off value for NLR as 3.9 (31).

Upon conducting a univariate analysis, we observed that the factors associated with a probability of higher NLR and dNLR were similar and included 51–75 years of age, >75 years, ICU stay, invasive ventilation, death, and third wave of the pandemic. The likelihood of a high NLR was associated with the second wave of the pandemic, while a high dNLR was correlated with the male gender as well. Previously, Haifeng Hu et al. deduced through a univariate logistic regression analysis that NLR and the age, comorbidity, hypertension, lymphocyte count, NLR, albumin, and CRP were associated with the disease severity of COVID-19 (32). Meanwhile, Ding et al. established that the NLR index positively correlated with the length of hospital stay and has a role in predicting the prognosis of disease for patients with COVID-19 (33). Djaharuddin et al. showed NLR’s significant predictive value and as a significant risk factor affecting severe disease incidence. The authors also found that according to NLR and age stratification, the occurrence of severely ill was associated with NLR ≥3.13 and aged ≥50 years old was 50%, and 9.1% in age ≥50 and NLR <3.13 patients (34). Our multivariate analysis divulged that 51–75 years, >75 years of age group, ICU stay, death, and third wave of the pandemic were independently associated with higher NLR and dNLR. A retrospective study from Wuhan revealed through multiple logistic regression analyses that NLR and acute myocardial injury were independently and negatively associated with death in patients with severe COVID-19. The study concluded that the risk of death increases by 5.7% for every one-unit increase in NLR (26).

A retrospective study conducted in China found the initial NLR to be significantly lower in survivors as compared to the deceased patients (35). In a recent analysis by Moradi et al., a high NLR was correlated with an increased risk of 1-month mortality (36). Tatum et al. created Kaplan–Meier curves using established cut-off points for hospital days 2 and 5 and revealed that the differences in survival for patients with COVID above the stated NLR cutoff value compared to those below the cutoff examined were highly statistically significant for each day examined (28). Another retrospective study evaluating COVID-19 infected individuals from the first and second waves of the pandemic found an elevated NLR of ≥3.13 in 87.18% of the patients and found NLR to be the most significant factor affecting the severe illness incidence, with a significant predictive value (34). Haifeng Hu et al. indicated through their Kaplan–Meir curve analysis with log-rank tests that venerable age (≥60 years old), comorbidity, hypertension, lymphopenia, hypoalbuminemia, elevated NLR, and CRP could hinder the recovery and discharge of patients (32).

In addition, NLR was also found to correlate with other markers of inflammatory response like CRP and D-dimer, while dNLR was found to be linked with CRP, PCT, and D-dimer in the survival analysis. Ye et al. also found a strong significant correlation between D-Dimer, NLR, and other markers (35). Fu et al. attempted to find COVID-19 markers from conventional hematological examinations and found NLR and D-dimer levels showed superior performance not only on admission but also on subsequent different days after admission (8). Yang et al. and Sun et al. examined some hematological indices in patients with COVID-19 and found that NLR, PLR, and monocyte-to-lymphocyte (MLR) values were significantly higher in severe patients than in non-severe patients (33). The blood urea nitrogen (BUN)/creatinine (Cr) ratio has also been evaluated as a marker and Fesih et al. found NLR and BUN/Cr to be independent predictors for disease severity (28). Multiple studies have corroborated NLR’s to be an independent risk factor for severe COVID-19 and in combination with other markers too such as CRP, it can be a reliable predictor of COVID-19 severity (37). We speculated dNLR to be a valid prognostic indicator too, as it had only minor differences in our analysis with NLR, such as the former being higher in males. Both NLR and dNLR had similar accuracy overall, however, our results revealed dNLR to have shown slightly higher sensitivity at optimum cut-off correlating with the higher negative likelihood ratios shown by NLR. The odds of mortality on multivariate analysis were higher for dNLR than for NLR. We find both markers to be advantageous, with the two outweighing each other in various key comparisons.

Neutrophil to lymphocyte ratio has been more widely researched and most studies elucidate its importance as it shows the balance between neutrophil count and lymphocyte count and, consequently the equilibrium between the severity of inflammatory response and the immune function of the body (23). Hence, an inadequate immune function can be assessed through the NLR value and can help identify patients that need immediate attention as they might have a poor prognosis and even a risk of death (35). Therapeutic intervention can also be administered through surveillance with NLR and measure immunosuppression treatments like methylprednisolone and cell normalization in patients who have recovered or are recovering (13), antagonists of cytokines or blockers of the complement system (13) based on the phase of the illness. Recognizing patients undergoing inflammation helps to direct the curative intent on the inflammation rather than the viral replication (13). A challenge posed is determining the appropriate threshold for NLR and dNLR. In addition, the time elapsed since the onset of COVID-19 symptoms may affect NLR (38), hence, taking into account that these ratios can be influenced by various factors, questioning their routine use. Nonetheless, when considering the time when the patients complained of fever, dry cough, dyspnea, chest distress, and other symptoms as day 0, changes and trends in CBC results of moderate, severe, and critical patients were analyzed, it was extrapolated that NLR was the most stable parameter, with the moderate group having results under NLR value 5.92 and the severe group having results over 5.92 (39).

Neutrophil to lymphocyte ratio and dNLR are simple, cost-effective markers for predicting COVID-19 outcomes. With a strain on resources in the current crisis, risk stratification with any of these two parameters can help in better allocation and monitoring of the progression of the disease (13) plausibly improving outcomes with timely intercession. Our study has provided robust data ranging from the first three waves of COVID-19, and these results can be employed when inspecting multiple approaches for laboratory tests and ascertaining which markers to use decisively. This study is not without its limitations. This research was conducted at a single-center hospital in a retrospective manner. Due to the retrospective nature of the study, confounding factors may have also affected the outcomes. The data collected were of patients who were admitted to the hospital with moderate to severe symptoms, data were not collected from patients with mild symptoms who were deemed fit to be treated at home. Also, during the hospitalization of the participants factors that may have affected the prognosis of the patients were not taken into account. As described previously, neutrophils and lymphocytes account for 96% of total leukocyte count, thus the changes of NLR and dNLR may be parallel, and the results might have similar accuracy to predict various outcomes in patients with COVID-19 due to random (statistical) noise within the data. Therefore, it is imperative to have a linear relationship between NLR and dNLR across all the cases. Lastly, collider bias can be introduced by associations between two or more variables that affect the likelihood of an individual outcome, such as mortality or ICU stay, hence distorting associations between these variables.

## Conclusion

Although the study did not include any longitudinal association of these markers with the hospital course, and further comparisons were not able to demonstrate significant differences among the two markers, the current study concludes that both markers are equally reliable and sensitive to predicting in-hospital outcomes of patients with COVID-19. Early detection and predictive analysis of these markers can help physicians assess risk and manage patients more effectively as the severity of these markers increases in subsequent waves.

## Data availability statement

The raw data supporting the conclusions of this article will be made available by the authors, without undue reservation.

## Ethics statement

The studies involving human participants were reviewed and approved by the Dow University Hospital. The ethics committee waived the requirement of written informed consent for participation.

## Author contributions

FY, RM, and MuA: conceptualization. IY, MuA, MoA, and MJ: data curation. HN and MuA: formal analysis. FY, MT, and HN: investigation and project administration. FY, RM, and HN: methodology. HM, MoA, and MuA: resources. HN and QM: software. FY and HM: validation. MuA, QM, and MJ: visualization. HN, UN, MG, and MuA: writing—original draft. FY, MG, MoA, and UN: writing—review and editing. All authors contributed to the article and approved the submitted version.

## Conflict of interest

The authors declare that the research was conducted in the absence of any commercial or financial relationships that could be construed as a potential conflict of interest.

## Publisher’s note

All claims expressed in this article are solely those of the authors and do not necessarily represent those of their affiliated organizations, or those of the publisher, the editors and the reviewers. Any product that may be evaluated in this article, or claim that may be made by its manufacturer, is not guaranteed or endorsed by the publisher.

## Supplementary material

Supplementary Figure 1 Multivariable regression for in-hospital outcomes with elevated neutrophil to lymphocyte ratio (NLR) and derived NLR (dNLR).

Supplementary Figure 2 Survival curves for in-hospital outcomes with elevated neutrophil to lymphocyte ratio (NLR) and derived NLR (dNLR).
