# Supplementary figures and images for: Comparative analysis of neutrophil to lymphocyte ratio and derived neutrophil to lymphocyte ratio with respect to outcomes of in-hospital coronavirus disease 2019 patients: A retrospective study
Source: Front Med (Lausanne). 2022 Jul 22;9:951556. doi: 10.3389/fmed.2022.951556 (PMC9354523; doi:10.3389/fmed.2022.951556)

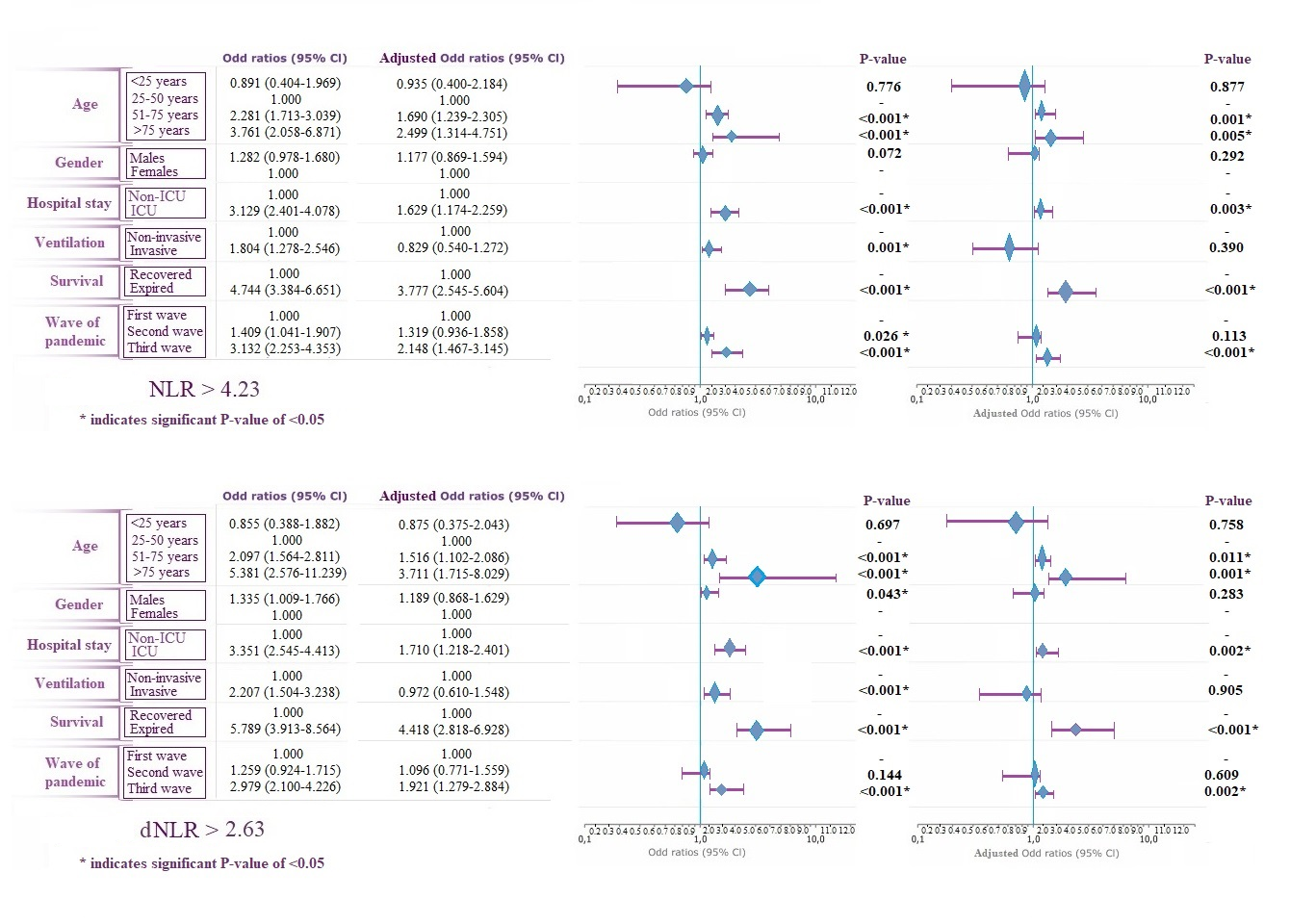

Supplement: Supplementary Figure 1 — Multivariable regression for in-hospital outcomes with elevated neutrophil to lymphocyte ratio (NLR) and derived NLR (dNLR). [file Image_1.TIFF]

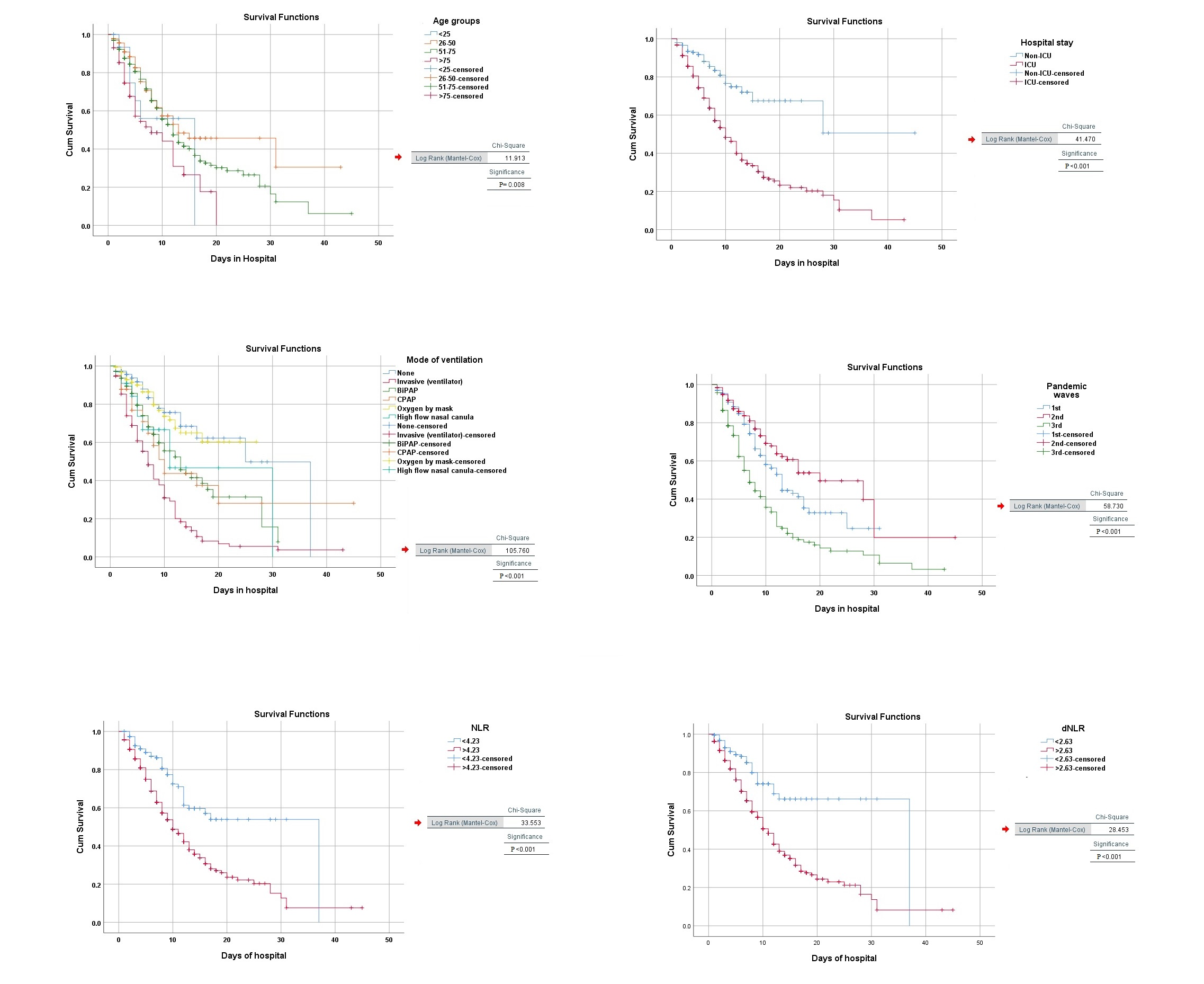

Supplement: Supplementary Figure 2 — Survival curves for in-hospital outcomes with elevated neutrophil to lymphocyte ratio (NLR) and derived NLR (dNLR). [file Image_2.TIFF]
